# Predictors of Mortality in Scrub Typhus Infection Requiring Intensive Care Admission in Tertiary Healthcare Centre of Nepal

**DOI:** 10.1155/2018/4867958

**Published:** 2018-06-03

**Authors:** Shital Adhikari, Ramesh Sharma Poudel, Shakti Shrestha, Praves Lamichhane

**Affiliations:** ^1^Pulmonology and Critical Care Unit, Department of Internal Medicine, Chitwan Medical College Teaching Hospital, Chitwan, Nepal; ^2^Hospital Pharmacy, Chitwan Medical College Teaching Hospital, Chitwan, Nepal; ^3^Department of Pharmacy, Shree Medical and Technical College, Chitwan, Nepal; ^4^Faculty of Science, University of Sydney, Sydney, NSW, Australia

## Abstract

**Introduction:**

This study aimed to explore the predictors of mortality from scrub typhus infection in patients requiring intensive care unit (ICU) admission.

**Materials and Methods:**

A retrospective study was conducted on 120 patients with serum ELISA IgM positive for scrub typhus (optical density ≥ 0.5) admitted at the medical ICU of Chitwan Medical College Teaching Hospital between April 2016 and September 2017. Data was extracted from patient medical records and electronic database of the hospital. The outcome measurement was mortality (Yes/No) due to the infection. A multivariate binary logistic regression analysis (*p* < 0.10) using potential variables from bivariate analysis (*p* < 0.25) was adjusted to predict the mortality.

**Results:**

The mortality rate was 20% (24/120). Factors associated with mortality, as found using bivariate analysis, were heart rate > 100/minute (*p* < 0.001), systolic blood pressure < 90 mmHg (*p* = 0.025), diastolic blood pressure < 60 mmHg (*p* = 0.032), serum creatinine > 1.4 mg/dl (*p* < 0.001), acute kidney injury requiring dialysis (*p* = 0.029), acute respiratory distress syndrome (*p* < 0.001), and shock requiring vasopressor (*p* < 0.001). Regression analysis showed age (odds ratio [OR] = 1.063; 95% CI = 1.010–1.118; *p* = 0.019) and serum creatinine (OR = 1.063; 95% CI = 1.010–1.118; *p* = 0.019) as significant predictors of poor outcome.

**Conclusion:**

Older age and high serum creatinine were found to be independent predictors of poor outcome in patients with scrub typhus admitted in medical ICU.

## 1. Introduction

Scrub typhus is a reemerging zoonotic bacterial infection in the “tsutsugamushi triangle” region of South and Southeast Asia, the Asian Pacific Rim, and Northern Australia [1–5]. It is an acute, febrile illness caused by* Orientia* (formerly Rickettsia)* tsutsugamushi*, an obligate intracellular Gram-negative bacterium [6]. It was estimated that about one million people are affected with scrub typhus annually [7]. It is being recognized as an important cause of acute undifferentiated febrile illness in recent times. Though it was first reported in Nepal in early 1981 (19/188, 10.10%) [8], it was not identified till 2004, when a study in Patan Hospital, Kathmandu, reported that 3.2% (28/876) of patients suspected for typhoid fever had serology positive for scrub typhus [9]. Another study in 2007 also reported the presence of scrub typhus in 22% (*n* = 103) of patients with fever [10]. Three months after the devastating earthquake in Nepal (August 2015), scrub typhus was officially confirmed as having magnitudes of fatal outbreak [5]. Moreover, Chitwan, a district in the southern part of the country, experienced an outbreak of the disease in 2015, causing several morbidities and mortalities. Despite this, it is still one of the underrecognized and neglected diseases in the world, including Nepal [5, 11].

Studies in Nepal have reported mortality rate due to scrub typhus ranging from 1.7% to 7.92% [5, 12, 13]. A systematic review reported a median case fatality rate of 1.4% (range 0–33.3%) in treated patients with scrub typhus infection [14]. Studies from different parts of the world reported several predictors for mortality in patients with scrub typhus infection [15–19]. Similarly, a previous study from Nepal reported that development of shock, presence of acute kidney injury (AKI) at the time of admission, pneumonia, and requirement of intensive care unit (ICU) were predictors of mortality requiring hospital admission [13]. Moreover, a study in tertiary care teaching hospital of India reported that APACHE-II score and duration of fever were independent predictors of mortality in scrub typhus infection requiring intensive care admission [20]. However, there was a paucity of data on predictors of mortality in scrub typhus infection, especially requiring intensive care admission, from Nepal. This study aimed to find out the predictors of mortality in scrub typhus infection requiring intensive care admission in tertiary healthcare centre of Nepal.

## 2. Materials and Methods

This is a retrospective study including patients with scrub typhus infection aged 16 years or more admitted in Medical Intensive Care Unit (MICU) of Chitwan Medical College Teaching Hospital (CMCTH) between April 2016 and September 2017. CMCTH is located in the southern part of Nepal and is a 650-bedded multispecialty hospital with seventeen beds in MICU. The ethical approval of this study was obtained from Institutional Review Committee of Chitwan Medical College (ref. number CMC-IRC: 2074/075: 35) and a confidentiality agreement with Chitwan Medical College was signed by all researchers using the medical records of patients and electronic dataset. Medical records of the patients with serum ELISA IgM positive for scrub typhus (optical density ≥ 0.5), using ELISA kit manufactured by InBios International Inc., USA, were reviewed for the study. Coinfected patients with dengue, influenza, malaria, leptospirosis, and typhoid fever were excluded. Sociodemographic information: age and sex; clinical features: duration of fever, signs and symptoms, comorbidities, heart rate (HR), systolic blood pressure (SBP), and diastolic blood pressure (DBP); organ dysfunctions; and complications were retrieved from patient medical records. Similarly, electronic database was used to collect the laboratory information that included hemoglobin level, white blood cell count (WBC), platelets count, bilirubin level, total protein level, albumin level, transaminases levels, and serum creatinine level. The outcome measurement was mortality (Yes/No).

Statistical analysis was performed by using IBM SPSS version 20 (IBM Corporation, Armonk, NY, USA). Normality was confirmed using Shapiro-Wilk test. Descriptive statistics were performed for all study variables. Chi-square and Mann–Whitney *U* tests, where appropriate, were performed for bivariate analysis to determine the level of association with outcome measurement. A multivariate binary logistic regression analysis (*p* < 0.10) using potential variables from bivariate analysis (*p* < 0.25) was adjusted to predict the mortality. For all statistical analysis, *p* < 0.05 was considered as statistically significant.

## 3. Results

Out of 120 patients, 42 (35%) were males. The median [interquartile range (IQR)] age was 41 (28.8) years. Majority of the patients had fever for less than seven days (64, 53.3%); heart rate more than 100 beats per minute (84, 70.0%); hemoglobin level less than 12 mg/dl (105, 87.5%); WBC count more than 11000 per mm^3^ (75, 62.5%); platelets count less than 100000 per mm^3^ (91, 75.8%); total protein level less than 6 g per dl (78, 65.0%); albumin level less than 3.5 g per dl (93, 77.5%); and shock requiring vasopressor (70, 58.3%). Fifty-five patients (45.8%) had acute respiratory distress syndrome (ARDS) and two-fifths patients had bilirubin level more than 1.5 mg/dl. Similarly, dialysis was performed in seven patients (5.8%). Most common complication was pneumonia (27, 22.5%). Others documented complications were meningoencephalitis (13, 10.8%), myocarditis (8, 6.7%), gastrointestinal bleeding (2, 1.7%), and arrhythmia (1, 0.8%). Of 120 patients, 96 (80.0%) patients recovered after treatment, while others (24, 20.0%) died (Table 1).

**Figure 1:**
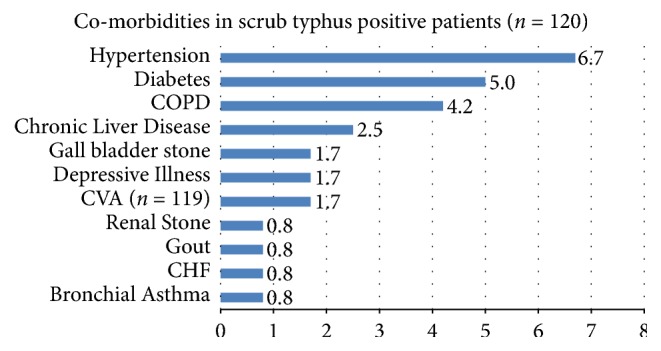
Comorbidities in scrub typhus positive patients. COPD: chronic obstructive pulmonary disease; CVA: cardiovascular accident; and CHF: congestive heart failure.

Most common comorbidities documented in our study was hypertension (8, 6.7%), followed by diabetes (6, 5.0%) and COPD (5, 4.2%). Details of other comorbidities are depicted in Figure 1.

**Figure 2:**
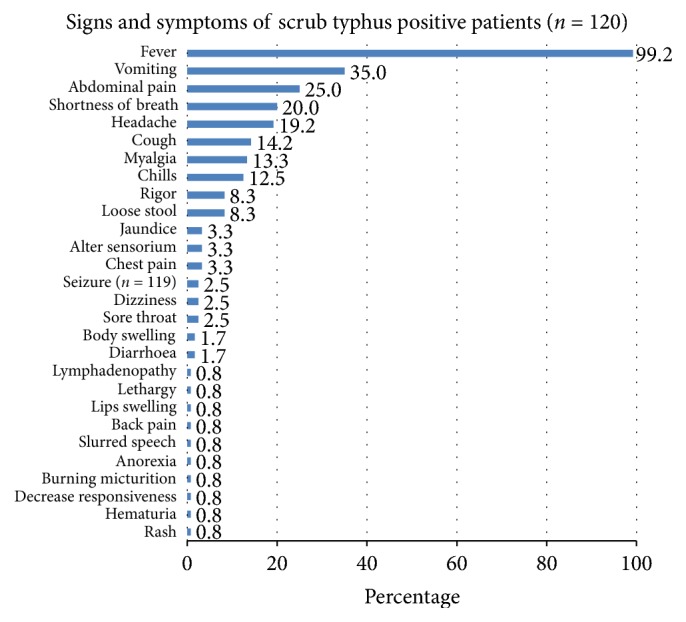
Signs and symptoms of scrub typhus positive patients.

The signs and symptoms of patients as seen in our study have been illustrated in Figure 2. Almost all the patients (119, 99.2%) had fever. Other chief complaints were vomiting (42, 35%), abdominal pain (30, 25%), shortness of breath (24, 20.0%), and headache (23, 19.2%).

Bivariate analysis demonstrated that statistically significant factors that were associated with mortality due to scrub typhus were HR more than 100 beats per minute (*p* < 0.001); SBP less than 90 mmHg (*p* = 0.025); DBP less than 60 mmHg (*p* = 0.032); serum creatinine level more than 1.4 mg per dl (*p* < 0.001); AKI requiring dialysis (*p* = 0.029); ARDS (*p* < 0.001); and shock requiring vasopressor (*p* < 0.001) (Table 2).

Table 3 shows the model, developed from multivariate logistic regression analysis of explanatory variables [*p* < 0.25 from Table 2: age (years), duration of fever (days), HR (>100/minute), SBP (<90 mmHg), DBP (<60 mmHg), WBC count (>11000 per mm^3^), platelets count (<100000 per mm^3^), bilirubin level (>1.5 mg/dl), total protein level (<6 g/dl), serum creatinine level (>1.4 mg/dl), AKI requiring dialysis, ARDS, shock requiring vasopressor, and meningoencephalitis], used to predict mortality of patients with scrub typhus. The model shows that age and creatinine were statistically significant predictors of mortality of ICU-admitted patients with scrub typhus infection. The odds of mortality for patients with scrub typhus admitted in ICU were higher for older patients and higher serum creatinine level. The model depicts that one year increase in age increases the odds of mortality by 1.063 times (*p* = 0.019; 95% CI = 1.010 to 1.118). However, those with serum creatinine level less than 1.4 mg/dl have 0.089 times lower odds of mortality compared to those with serum creatinine level more than 1.4 mg/dl (*p* = 0.004; 95% CI = 0.017 to 0.463).

## 4. Discussion

Scrub typhus is considered as one of the neglected diseases in the world, including Nepal [5, 11], with median case fatality rate of 1.4% (range 0–33.3%) [14]. Overall, mortality rate in our study was 20%, which is lower than that reported by a similar study in India (24.1%) [20]. Such high mortality rate might be due to the delayed arrival in hospitals. As a result, patients might have developed multiple organ failure before initiating therapy, primarily doxycycline. Moreover, clinical failure of doxycycline has also been reported [21]. However, these issues need further exploration before reaching conclusion in our setting.

Difference has been observed between median ages of the patients with respect to outcomes in this study. However, this difference was not statistically significant in bivariate analysis. Similar results were observed in other studies [16, 17, 22], while some studies have indicated that increasing age is associated with death [20, 23, 24]. On multivariate analysis, age was observed to be a statistically significant predictor of mortality in our study, suggesting that increase in one year of age increases the odds of mortality by 1.063. Outcome was not associated with the sex of the patients in this study, and this finding is consistent with other studies [16, 20, 22, 24].

Studies have shown an association between duration of illness and mortality, indicating that shorter duration leads to poor outcome [17, 20]. It has also been reported that shorter duration of illness increases the risk of mortality [Relative Risk (RR) = 0.82 (95% CI = 0.67–0.99) to RR = 0.9 (95% CI = 0.82–0.96)] [15, 17] and lesser duration of fever increases the odds of mortality (OR = 0.75; 95% CI = 0.6–0.9) [20]. However, study conducted by Peter et al. did not observe such association [22], and our study also found similar results. Moreover, we were unable to evaluate the effect of appropriate antibiotic on the mortality prior to treatment. HR (>100/minute), SBP (<90 mmHg), and DBP (<60 mmHg) were associated with poor outcomes in our study; however, these effects were not observed on multivariate analysis. Although bivariate analysis has shown an association of mortality with HR (>100/minute) [19] and SBP (<90 mmHg) [19, 24], a study conducted by Thipmontree et al. in Thailand has found no significant association with HR [24]. Furthermore, studies demonstrate that SBP (<90 mmHg) increases the risk of mortality by 1.7 (95% CI 0.43–6.8) to 2.8 (95% CI 1.5–5.3) times [15, 17].

Our study did not show a significant association between hemoglobin level (<12 mg/dl) and mortality, which is similar to the findings of other studies [16, 17, 19, 23], though a study reports a different finding [24]. Similarly, mortality was not significantly influenced by WBC count (>11000 per mm^3^) in this study. Although a similar finding has been documented [24], most studies found the role of WBC count on mortality [16, 17, 19, 23]. Evidences suggest that increase in leucocyte count increase the RR of mortality by nearly one time [15, 17]. In sharp contrast to this, a study done by Varghese et al. demonstrated that the effect of WBC count (<11,000/mm^3^) on RR of mortality of patient infected with scrub typhus is not significant [18]. Furthermore, our study also showed no significant association between platelets count (<100000 per mm^3^) and mortality, which is similar to the results reported by other studies in other countries [16, 19]. In contrast to these findings, several studies conducted in Indian settings reported increased risk of mortality in patients with lower platelets count compared to those with higher platelet count [15, 17, 25]. Similarly, bilirubin level (>1.5 mg/dl) was not associated with mortality in this study. Several studies have also demonstrated no significant association between bilirubin level and mortality [16, 17, 19]. However, one study demonstrated that bilirubin level (>1.5 mg/dl) significantly increased the RR of mortality by 9.28 (1.48–58.5) times [18]. Similarly, another study found serum bilirubin level ≥ 2.5 mg/dl increased the risk of mortality by 3.1 (1.7–5.4) times in comparison to level lower than 2.5 mg/dl [15]. Likewise, our study did not find a significant association between total protein level (<6 g/dl) and mortality, which is similar to other studies [17, 19]. There have been inconsistent reports in the literature with respect to the effect of albumin level on mortality [16, 17, 19, 23, 24], and our result suggests no significant association of serum albumin level with mortality. Transaminase level (>120 IU/l) was not associated with mortality in our study. Other studies also demonstrated similar findings [16, 17, 24]. However, a study in India reported that patients with comparatively higher level of aspartate aminotransferase (AST) and alanine aminotransferase (ALT) were associated with mortality [15, 23, 25]. Our study demonstrated significant association of serum creatinine level (>1.4 mg/dl) with mortality. Serum creatinine level > 1.4 mg/dl was found to be an independent predictor of mortality, suggesting that the odds of mortality in patients with serum creatinine level > 1.4 mg/dl are 0.098 times higher as compared to those with serum creatinine level ≤ 1.4 mg/dl (*p* = 0.098; 95% CI = 0.016 to 0.580). This finding is consistent with the previous finding reported in other studies [18, 24, 26, 27]. Similarly, a study from Nepal also reported AKI at the time of admission to be one of the predictors of mortality, but without odds ratio [13]. Furthermore, another study reported that creatinine level > 2 mg/dl independently increased odds for mortality by 3.5 (1.7–7.1) times in patients infected with scrub typhus [15]. Similarly, study by Sivarajan et al. demonstrated that serum creatinine level > 1.5 mg/dl is a predictor of mortality (OR = 18.03, 95% CI = 1.38 to 235.1) [25].

Our study showed that AKI requiring dialysis was associated with mortality, which is consistent with the finding of a previous study [20]. Moreover, a study has also reported the need for dialysis as an independent predictor for mortality [22]. This study demonstrated a significant association between ARDS and mortality; however, it was not independently associated with mortality. However, previous studies reported that ARDS to be an independent predictor for mortality [19, 22]. Similarly, hypotension requiring vasoactive agents [15, 17], or shock [19], has also been known to be an independent predictor of mortality. But, we did not observe such results in multivariate analysis. Presence of meningoencephalitis, gastrointestinal bleeding, pneumonia, arrhythmias, and myocarditis was not associated with mortality in this study, probably due to a lesser number of patients suffering from these complications. Others studies also reported CNS dysfunction [15], APACHE-II score [16, 20, 22], metabolic acidosis [19], altered sensorium [19, 27], pneumonia [13], procalcitonin [22], and absence of eschar [16] as independent predictors for mortality. SOFA score [22], von Willebrand factor [28], high mobility group box 1 (HMGB1) [28], cytokine (interleukin-8) [29], endothelial marker, and macrophage marker [30] are also associated with poor outcome.

## 5. Conclusions

Older age and high serum creatinine were found to be independent predictors of poor outcome in patients with scrub typhus admitted in medical ICU of Chitwan Medical College Teaching Hospital. Morbidity and mortality associated with scrub typhus can be reduced significantly by early recommended empiric therapy with doxycycline in clinically suspected cases of scrub typhus.

## Figures and Tables

**Table 1:** Baseline characteristics (*n* = 120).

Characteristics	*n* (%)
^a^Age in years	41 (28.8)
Sex (female)	78 (65.0)
Duration of fever	
<7 days	64 (53.3)
7–14 days	51 (42.5)
15–21 days	5 (4.2)
HR (>100/minute)	84 (70.0)
SBP (<90 mmHg)	58 (48.3)
DBP (<60 mmHg)	59 (49.2)
Haemoglobin level (<12 mg/dl)	105 (87.5)
WBC count (>11,000/mm^3^)	75 (62.5)
Platelets count (<100,000/mm^3^)	91 (75.8)
Bilirubin level (>1.5 mg/dl)	48 (40.0)
Total protein level (<6 gm/dl)	78 (65.0)
Albumin level (<3.5 gm/dl)	93 (77.5)
Transaminase level (>120 IU/l)	65 (54.2)
Serum creatinine level (>1.4 mg/dl)	45 (37.5)
ARDS	55 (45.8)
Shock requiring vasopressor	70 (58.3)
AKI requiring dialysis	7 (5.8)
Meningoencephalitis	13 (10.8)
Gastrointestinal bleeding	2 (1.7)
Pneumonia	27 (22.5)
Arrhythmia	1 (0.8)
Myocarditis	8 (6.7)
Outcomes	
Recovered	96 (80.0)
Death	24 (20.0)

^a^Median [interquartile range (IQR)], ARDS: acute respiratory distress syndrome; AKI: acute kidney injury; DBS: diastolic blood pressure; HR: heart rate; SBP: systolic blood pressure; WBC: white blood cell.

**Table 2:** Bivariate analysis of outcome measures of scrub typhus (*n* = 120).

Characteristics	Mortality		*p* value
	No	Yes	
	*n* (%)	*n* (%)	
^a^Age in years	40.0 (30.8)	46.5 (31.8)	0.114
Sex (female)	62 (79.5)	16 (20.5)	1.000
Duration of fever			
<7 days	48 (75.0)	16 (25.0)	0.149
7–14 days	43 (84.3)	8 (15.7)	
15–21 days	5 (100.0)	0	
HR (>100/minute)	61 (72.6)	23 (27.4)	<0.001^*∗∗*^
SBP (<90 mmHg)	41 (70.7)	17 (29.3)	0.025^*∗*^
DBP (<60 mmHg)	42 (71.2)	17 (28.8)	0.032^*∗*^
Hemoglobin level (<12 mg/dl)	83 (79.0)	22 (21.0)	0.733
WBC count (>11000 per mm^3^)	57 (76.0)	18 (24.0)	0.239
Platelets count (<100000 per mm^3^)	70 (76.9)	21 (23.1)	0.220
Bilirubin level (>1.5 mg/dl)	34 (70.8)	14 (29.2)	0.069
Total protein level (<6 g/dl)	59 (75.6)	19 (24.4)	0.165
Albumin level (<3.5 g/dl)	72 (77.4)	21 (22.6)	0.299
Transaminase level (>120 IU/l)	52 (80.0)	13 (20.0)	1.000
Serum creatinine level (>1.4 mg/dl)	26 (57.8)	19 (42.2)	<0.001^*∗∗*^
AKI requiring dialysis	3 (42.9)	4 (57.1)	0.029^*∗*^
ARDS	31 (56.4)	24 (43.6)	<0.001^*∗∗*^
Shock requiring vasopressor	46 (65.7)	24 (34.3)	<0.001^*∗∗*^
Meningoencephalitis	8 (61.5)	5 (38.5)	0.113
GI bleeding	1 (50.0)	1 (50.0)	0.361
Pneumonia	24 (88.9)	3 (11.1)	0.299
Arrhythmia	1 (100.0)	0 (0)	1.000
Myocarditis	4 (50.0)	4 (50.0)	0.500

^a^Median [interquartile range (IQR)], ARDS: acute respiratory distress syndrome; AKI: acute kidney injury; DBS: diastolic blood pressure; HR: heart rate; SBP: systolic blood pressure; WBC: white blood cell. ^*∗*^Significant at *p* < 0.05 and ^*∗∗*^significant at *p* < 0.001.

**Table 3:** Regression model for predicting mortality of ICU patients with scrub typhus (*n* = 120).

Variables	*β* (SE)	*p* value	Odds ratio	95% CI EXP (B)	
				Lower	Upper
Age	0.061 (0.026)	0.019	1.063	1.010	1.118
Serum creatinine (>1.4 g/dl)	−2.417 (0.840)	0.004	0.089	0.017	0.463

Model *χ*^2^ = 76.137 (*p* < 0.001); −2 Log likelihood = 43.960; Cox & Snell *R*^2^ = 0.470; Nagelkerke *R*^2^ = 0.743; Hosmer and Lemeshow *p* = 0.986.

## Data Availability

The datasets used and/or analyzed during the current study are available from the corresponding author on reasonable request.
